# *In vitro* and *in vivo* antiplasmodial activity and cytotoxicity of extracts from *Vernonia amygdalina* Del. Leaves

**DOI:** 10.1186/1475-2875-9-S2-P30

**Published:** 2010-10-20

**Authors:** Ehimwenma S Omoregie, Anirban Pal, Mahendra P Darokar, Debabrata Chanda, Brijesh Sisodia

**Affiliations:** 1 Department of Biochemistry, Faculty of Life Sciences, University of Benin, PMB 1154, Benin City, Nigeria; 2 In vivo Animal Testing Facility, Central Institute of Medicinal and Aromatic Plants (CIMAP), Lucknow, India; 3 In vitro Cell Culture Laboratory, Genetic Resources and Biotechnology Department, Central Institute of Medicinal and Aromatic Plants (CIMAP), Lucknow, India

## Background

This study evaluated the *in vitro* and *in vivo* antiplasmodial activity of extracts from *V. amygdalina* leaves. The plant was selected based on local claims on its efficacy as part of the treatment regimen in malarial infection in the south - western region of Nigeria.

## Materials and methods

Extracts of the plant (ethanolic, aqueous, and hydroethanolic (50:50) extracts) were prepared using standard procedures. Chemical profile of the extracts was performed through high performance thin layer chromatography (HPTLC) for quality control. The extracts were evaluated *in vitro* for antiplasmodial activity using a 3D7 chloroquine sensitive clone of NF-54 isolate of *Plasmodium falciparum.* The parasite growth inhibition was estimated based on the 48 hours microassay technique [1]. Cytotoxicity of these extracts was evaluated *in vitro* against non-cancerous *vero* cell lines (C-1008 kidney fibroblasts from African green monkey) by the neural red uptake method [2]. The *in vivo* antiplasmodial activity of the most active extract(s) was assessed based on the standard four days suppressive test on *P. berghei* (ANKA) infected male mice (six weeks old) of the Swiss strain [3].

## Results

Results from the *in vitro* study showed that the ethanolic extract of the plant leaves had the highest (p<0.05) antiplasmodial activity (IC_50_ = 9.83 ug/ml) and cytotoxicity (IC_50_ = 60.33) with moderate selectivity index of 6. 14 when compared with the other extracts. The ethanolic extract was also significantly active *in vivo* against *P. berghei* in a dose-dependent manner with maximum activity observed at 1000 mg/kg (% inhibition of 82.3 %). There was also a dose-dependent significant decrease (p<0.05) in some oxidative stress indices especially nitric oxide and malonaldehyde levels. The pro-inflammatory cytokines (TNF-α and IFN-γ) levels were also considerably low relative to control values.

## Conclusions

The results suggest that *V. amygdalina* possess moderate antiplasmodial activity both *in vitro* and *in vivo.* The immunomodulatory and antioxidant activities of this extract may be responsible for its antiplasmodial property. The study therefore confirms local claims on the use of the plant leaves as part of the treatment regimen in malarial infection.
